# Supplementary material for: Salt Stress Inhibits Photosynthesis and Destroys Chloroplast Structure by Downregulating Chloroplast Development–Related Genes in Robinia pseudoacacia Seedlings
Source: Plants (Basel). 2023 Mar 11;12(6):1283. doi: 10.3390/plants12061283 (PMC10054032; doi:10.3390/plants12061283)
Supplement: Supplementary file 1 [file plants-12-01283-s001.zip › plants-2262564-supplementary.pdf]

Supplementary Materials

Table S1. Primer pairs for real-time quantitative PCR.

Gene ID	F1 (5'-3')	F2 (5'-3')
RppsbA	ACTGCAATTTTAGAGAGACGCGAGA	AATACCTACTACAGGCCAAGC
RppsaB	AAGGCTTAGCTCAGGACC	CGCCAGGAGATTAAGAAC
RppsaA	AAGCATGGCTAAGTGATC	GTCCGCTGAATAGAAACA
RppsbD	CGGTCGTGACCAAGAAAC	CGGATAAAGCACCTAAAC
RpndhE	ATGCTCGAACATGTACTTGTTTTGA	AATCCGATAGCTGCTTCA
RpndhH	CAGAAGCAATAACCGTAAATGG	TCCAGAGCCTGCTGAATAA
RpropA	CGCTTTATTCTGTCTCCA	AGCCTTAGCCAATCTATC
RppcbC	CGGTCGTGACCAAGAAAC	CGGATAAAGCACCTAAAC
Rprps7	TCACGGCGAGGTACTGCAGAAAAAA	ACTCCCTTTGGCAGCATC
RppsaC	TCACATTCAGTAAAGATTTATGATA	GCCATAAATAAACCCGAACA
RpSOS1	CTCTGCCATCTTCTGTTC	CACTTCACCACTCCATTAG
RpNHX1	CCTATCTGAGTGGCATTCT	GCTTGGTAGTGATTCTTGAG
